# High Oxygen Exchange to Music Indicates Auditory Distractibility in Acquired Brain Injury: An fNIRS Study with a Vector-Based Phase Analysis

**DOI:** 10.1038/s41598-018-35172-2

**Published:** 2018-11-13

**Authors:** Eunju Jeong, Hokyoung Ryu, Joon-Ho Shin, Gyu Hyun Kwon, Geonsang Jo, Ji-Yeong Lee

**Affiliations:** 1 Department of Arts and Technology, Hanyang University, Seoul, 04763 Republic of Korea; 2 Division of Industrial Information Studies, Hanyang University, Seoul, 04763 Republic of Korea; 3 Graduate School of Technology and Innovation Management, Hanyang University, Seoul, 04763 Republic of Korea; 4 Department of Neurorehabilitation, National Rehabilitation Center, Ministry of Health and Welfare, Seoul, 01022 Republic of Korea

## Abstract

Attention deficits due to auditory distractibility are pervasive among patients with acquired brain injury (ABI). It remains unclear, however, whether attention deficits following ABI specific to auditory modality are associated with altered haemodynamic responses. Here, we examined cerebral haemodynamic changes using functional near-infrared spectroscopy combined with a topological vector-based analysis method. A total of thirty-seven participants (22 healthy adults, 15 patients with ABI) performed a melodic contour identification task (CIT) that simulates auditory distractibility. Findings demonstrated that the melodic CIT was able to detect auditory distractibility in patients with ABI. The rate-corrected score showed that the ABI group performed significantly worse than the non-ABI group in both CIT1 (target contour identification against environmental sounds) and CIT2 (target contour identification against target-like distraction). Phase-associated response intensity during the CITs was greater in the ABI group than in the non-ABI group. Moreover, there existed a significant interaction effect in the left dorsolateral prefrontal cortex (DLPFC) during CIT1 and CIT2. These findings indicated that stronger hemodynamic responses involving oxygen exchange in the left DLPFC can serve as a biomarker for evaluating and monitoring auditory distractibility, which could potentially lead to the discovery of the underlying mechanism that causes auditory attention deficits in patients with ABI.

## Introduction

Acquired brain injury (ABI) is damage to the brain that occurs due to certain events after birth^1^, including a traumatic injury to brain tissues as a result of an external mechanical force^2^ and/or stroke, which is an ischemic or hemorrhagic cerebrovascular accident^3^. These events result in permanent or temporary impairments in various functional behaviours, including movement, emotion, perception, and cognition^4–8^. Cognitive impairments are pervasive among patients with ABI with attention deficits being the most common^9–11^. Selective attention, in particular, is considerably affected in patients with ABI^12–14^ as they are more susceptible to distraction^4,15–17^.

Auditory attention seems more challenging than visual attention in patients with ABI^18,19^. For example, individuals with ABI are incapable of focusing their attention to relevant auditory information. They also have difficulty distinguishing target sounds from distracting sounds^20^, and this notion is supported by previous studies that have shown that attentional performance of ABI patients is decreased during auditory distraction^21–25^. Attenuated auditory processing is probably due to the complex nature of auditory environments, which requires the simultaneous processing of many features, such as locational, timbre, and semantic cues. This consequently affects higher cognitive functions, such as working memory and executive control^26^ leading to, among other things, such as difficulty with speech perception in noisy environments, which can have long-term negative socio-communicative consequences.

Various neurocognitive assessments are used to diagnose and identify cognitive impairments in patients with ABI (e.g., information processing speed, short-term, and long-term memory capacity^27,28^). Visual stimuli have been mostly employed in the tests, such as the Stroop Colour-Word test^29^, Trail Making Test A and B^30^ and Delis-Kaplan Executive Function System Colour-Word Interference Test^31^. Relatively less attention has been drawn to auditory modality. The previous studies focused on the sequential processing of auditory information^32–34^ by using auditory stimuli involving spoken words and/or numbers in their tasks [e.g., paced auditory serial addition test^35^, auditory Stroop test^36^, and digit span test^37^. Given that distractibility is a significant contributor to the attention deficits in patients with ABI^15–17^, the development of an auditory perception test is needed in order to simulate situations requiring selective attention against simultaneous distraction.

Musical scenes assimilate real-world auditory environments in which more than two sounds streams are present. According to Bigand *et al*.^38^ listening to two or more concurrent music streams (e.g., a melody with harmonic accompaniment or two melodies) activates different types of attention, such as selective alternating and divided attention. One of the musical features, instrumental timbres, provides a perceptual cue that aids segregating multiple streams and focusing on a relevant one, such as in speech perception in noise^39–42^. Moreover, multi-voice music listening tasks activate neural regions involved in different types of attention^43–46^. The regions include the primary sensory cortices, superior temporal sulcus, and frontal and parietal regions, which are similarly activated in attention tasks using both musical and visual stimuli^43,44,47–49^.

Several studies have employed melodic contours to specifically show cognitive decline in patients with ABI and mild cognitive impairment^50–53^. Jeong and colleagues^51,52^ have developed a music-based attention assessment to evaluate attentional function in individuals with moderate to severe traumatic brain injury (TBI). Their primary findings^52^ showed that the melodic contour identification task (CIT) can be used with high reliability for healthy adults and patients with TBI. A follow-up study^51^ demonstrated the construct validity of four different factors of attention. Recently, cognitive loads across melodic CITs were examined using functional near-infrared spectroscopy (fNIRS) and demonstrated that as CIT difficulty increased, cognitive loads increased accordingly^54^. Collectively, these results suggest that the melodic CIT can simulate the different types and levels of auditory attention and, thus, can be used to evaluate attention deficits following brain injury.

In this study, we employed fNIRS combined with an advanced topological method to examine cerebral hemodynamic changes involving oxygen exchange during a music-cognition task. The cerebral hemodynamic response is one of the neuropathological indicators of ABI. ABI-specific changes are characterized by an imbalance between oxygen supply and consumption, which appears as hypoxia and cerebral ischemia^55,56^. Cerebral ischemia and hypoxia are prevalent secondary complications of ABI that are associated with poor outcome in ABI survivors^57–60^. Thus, indices associated with oxygen metabolism have been considered important for making region-specific assessments and planning effective rehabilitation according to the recovery phase^58,61–65^.

Although several studies have employed fNIRS to demonstrate its applicability and efficacy in monitoring hemodynamic responses^66–70^ and to examine cognitive alteration in patients with ABI, they have yielded mixed results^71–73^. During a target detection task, Merzagora *et al*.^74^ observed significantly decreases in oxygenated hemoglobin (HbO_2_) in ABI patients as compared with healthy adults. Similarly, Kontos *et al*.^75^ and Hashimoto *et al*.^76^ have reported large decreases or inactivation of HbO_2_ in the ABI group during a series of cognitive tasks (e.g. word memory, digit-symbol substitution, and working memory tests). Conversely, Hibino *et al*.^77^ found an overall enhancement of HbO_2_, specifically in the lateral and medial frontal regions, in patients with ABI.

The incongruence of these studies was possibly due to the use of a single hemodynamic index, namely HbO_2_, as this is insufficient to accurately explain the cognitive and neuropathological changes following ABI. Therefore, it is necessary to include multiple indices, such as the difference between oxygenated and deoxygenated haemoglobin, and total amount of haemoglobin concentration in order to investigate the dynamic relationship among them. The purpose of the study was to empirically demonstrate auditory distractibility of patients with ABI as evidenced by alteration in cerebral hemodynamic changes. We employed fNIRS combined with a topological vector-based analysis method to address the existing controversies in fNIRS studies. Furthermore, novel stimuli and tasks using music, namely melodic CITs were utilized to simulate distractibility in a real-world auditory environment.

## Results

### Behavioral Responses

Table 1 shows the mean performances on the CIT for accuracy and reaction time for the ABI and non-ABI groups. The mean accuracy in the non-ABI group was 75% for CIT1 and was followed by CIT2 (48.4%). Accuracy was lowest (40.4%) when attention shift was required between two concurrent melodic contours (CIT3). A similar trend was found across the CITs for the ABI group (decreasing from 61.5% to 21.5%), but accuracy declined considerably more between CIT1 and CIT2 than it did in the non-ABI group. Reaction time was not sensitive enough to reveal the difference between CIT1 and CIT2 for both the ABI and non-ABI groups. The mean response time in the ABI group was highest in CIT1, which was followed by CIT2 and CIT3. CIT1 and CIT2 did not differ statistically in pairwise post hoc analysis with Bonferroni correction (*p* < 0.05). However, this was not the case in the non-ABI group.

**Table 1:** Descriptive statistics (accuracy & response time for the ABI and non-ABI groups).

Task	Accuracy (%)				Response Time (ms)			
	Non-ABI		ABI		Non-ABI		ABI	
	Mean	SD	Mean	SD	Mean	SD	Mean	SD
CIT1	73.70	0.29	61.54	0.27	6879.31	4284.49	13334.83	6472.22
CIT2	48.40	0.22	23.23	0.18	7159.07	4344.95	12718.14	6097.92
CIT3	40.40	0.22	21.46	0.14	6598.32	4011.18	9964.29	4566.20
Mean	54.17	0.24	35.41	0.20	6878.90	4213.54	12005.75	5712.11

**Figure 1:**
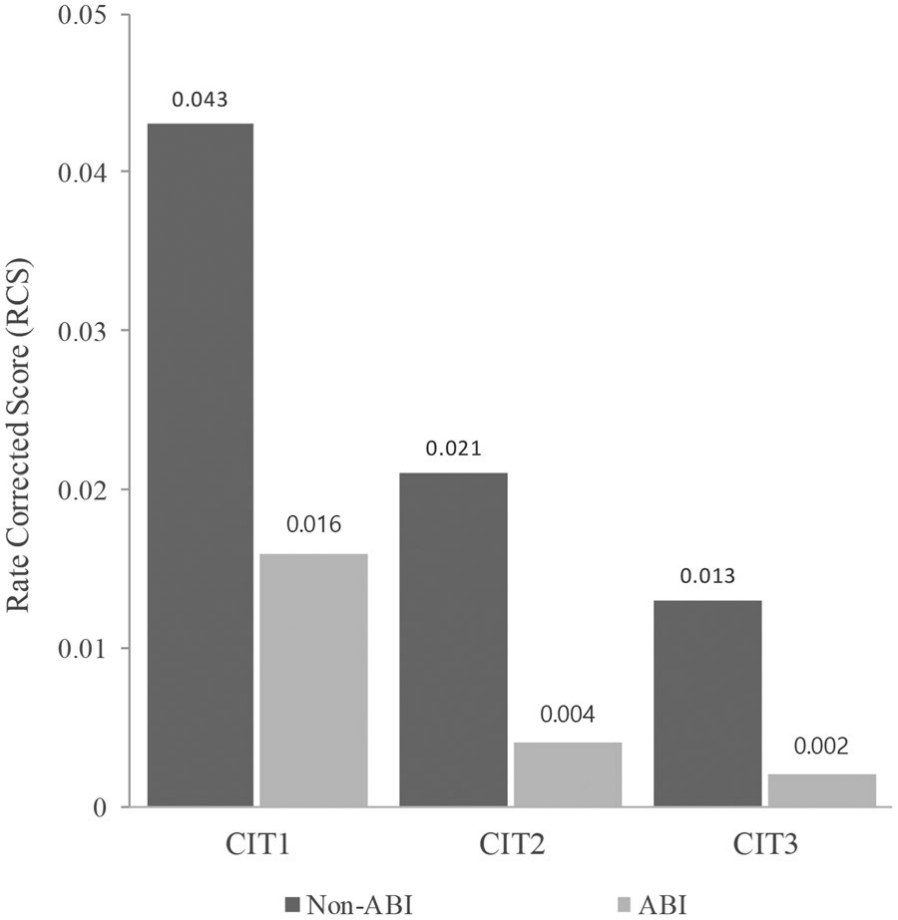
Changes in the rate-corrected score across CITs.

To illustrate the behavioral response trend, the RCS was calculated (Fig. 1), and a two-way mixed ANOVA [i.e. Group (ABI, non-ABI) × Task (CIT1, CIT2, and CIT3)] was performed. There was a significant main effect of Group [*F*_(1,31)_ = 12.671, *p* < 0.01] and Task [*F*_(2,62)_ = 35.643, *p* < 0.001], indicating that the overall performance was significantly better in the non-ABI group as compared with the ABI group and that performance worsened when the CIT was more difficult. In addition, the interaction between Group and Task was significant [*F*_(2,62)_ = 4.146, *p* < 0.05]. The pairwise post hoc comparison using a Bonferroni correction revealed that the ABI group performed significantly better in CIT1 than in CIT2 (*p* < 0.05), while the non-ABI group did better in CIT1 than in CIT2 (*p* < 0.001) and in CIT2 than in CIT3 (*p* < 0.05). These findings indicated that CIT1 and CIT2 were able to detect between- and within-group differences. CIT3 was thus excluded in the following hemodynamic response analysis.

### Hemodynamic Responses

**Figure 2:**
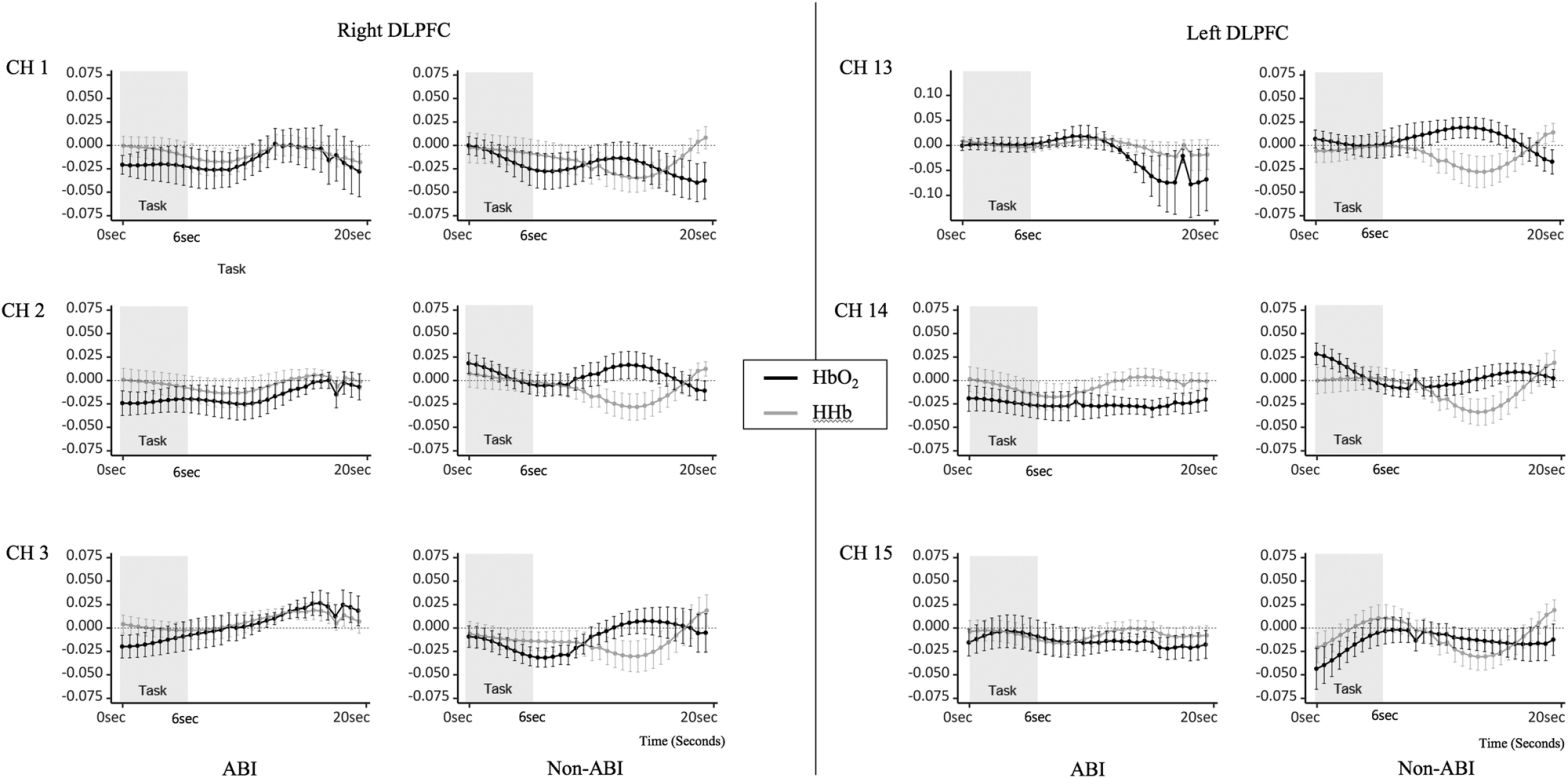
Examples of changes in HbO_2_ and HHb in the six fNIRS channels.

Descriptive statistics showed that HbO_2_ was generally less active in the ABI group, which differed from previous findings (Hibino *et al*., 2013) (see SI Appendix, Table 1 for details). In addition, the changes in the HbO_2_-HHb patterns differed between the groups. Figure 2 is examples of changes in oxygenated haemoglobin (HbO_2_) and deoxygenated haemoglobin (HHb) data for the ABI and non-ABI groups. The sample data set showed that decreases in HHb and increases in HbO_2_ were observed in the non-ABI group, while both HHb and HbO_2_ changed in the same direction in the ABI group (See SI Appendix, Tables 2 and 3 for details). These findings confirmed that vector-based phase analyses of both HbO_2_ and HHb were effective.

**Figure 3:**
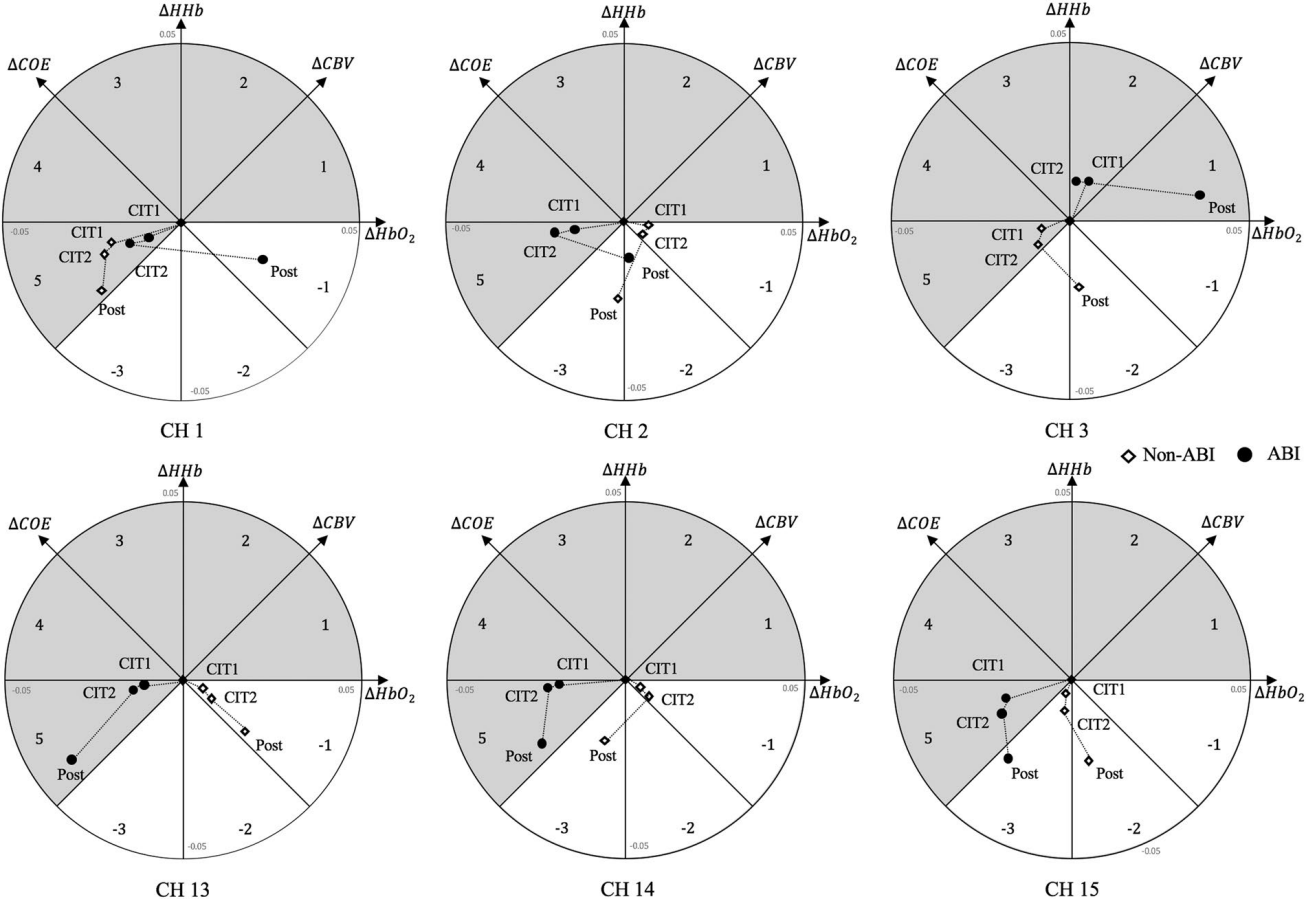
Trajectory of the vectors in the six fNIRS Channels.

With the vector-based phase analyses, we calculated ΔCOE, ΔCBV, angle *k* and scalar *L* and plotted the indices in a vector plan, which allowed for more insightful and efficient inspections of whether strong (or abnormal) oxygen metabolic demands were present (please see Methods for details). Figure 3 shows the relationship between oxygen demand and oxygen supply using k and L. The overall trend showed higher ΔCOEs in the ABI group than in the non-ABI group and lower ΔCBVs in the ABI group than in the non-ABI group. Generally, angle *k* tended to increase to 225° (phase 5) in the ABI group, while it decreased to −135° (phase 1) in the non-ABI group. The phasic information indicated that the non-ABI group was typically in phases 1 to 2 (hyperoxia/hyperemia and hyperoxia/ischemia, respectively), which indicated that the supply of HbO_2_ and total amount of blood were sufficient. However, the ABI group was in phases 4 to 5 (hypoxia/ischemia), which indicated that both the supply of HbO_2_ and total blood flow were insufficient. Exceptions were found in CH1 and CH2 only during the post-task when the cognitive demands were no longer present.

To confirm the abnormalities in the oxygen metabolic demands, the four indices were converted to a PRI analysis (see SI Appendix, Table 4 for details). To standardize the differences in the individual values, we subtracted the mean value during the pre-task from the mean values during the on-task and post-task. A two-way mixed ANOVA was performed using Group (ABI, non-ABI) × Task (pre-task, on-task, post-task) for each of the six channels. The CIT1 results indicated a significant main effect of Group on the PRI at CH13 *F*_(1,34)_ = 4.320, *p* < 0.05] and CH15 [*F*_(1,34)_ = 4.920, *p* < 0.05] and a significant main effect of Task on the PRI at CH1 [*F*_(2,68)_ = 4.830, *p* < 0.05], CH14 [*F*_(2,68)_ = 3.348, *p* < 0.05] and CH15 [*F*_(2,68)_ = 3.891, *p* < 0.05]. In addition, an interaction effect was found between Group and Task at CH15 [*F*_(2,68)_ = 3.620, *p* < 0.05]. A post hoc comparison revealed that the PRI was higher during the on-task than the pre-task in the ABI group (*p* < 0.05). The CIT2 results indicated a significant main effect of Group on the PRI at CH13 [*F*_(1,34)_ = 4.909, *p* < 0.05] and CH15 [*F*_(1,34)_ = 6.270, *p* < 0.05] and a significant main effect of Task on the PRI at CH1 [*F*_(2,68)_ = 4.496, *p* < 0.05], CH14 [*F*_(2,68)_ = 3.387, *p* < 0.05] and CH15 [*F*_(2,68)_ = 4.678, *p* < 0.05]. Most importantly, a significant interaction effect was found between group and task at CH15 [*F*_(2,68)_ = 3.520, *p* < 0.05]. A post hoc comparison revealed that the PRI was higher during the on-task than the pre-task in the ABI group (*p* < 0.01).

These findings showed that the PRI values were significantly higher in the ABI group than in the non-ABI group, indicating a greater level of oxygen exchange in the ABI group and during CIT1 and CIT2. The PRIs were significantly higher in CIT1 and CIT2 than the baseline, indicating increases in oxygen exchange during CIT performance. In particular, these changes were more prominent in the left than right DLPFC.

## Discussion

The present study attempted to use fNIRS technology in conjunction with a topological vector-based analysis method to assess cerebral hemodynamic changes underlying auditory dysfunctions in patients with ABI. Behavioral and hemodynamic responses were measured while individuals with ABI and without ABI performed melodic CITs. The RCS analysis showed that ABI-specific auditory distractibility was found in CIT1 and CIT2. In addition, cerebral hemodynamic changes during CIT1 and CIT2 showed a greater level of oxygen exchange in the ABI group than in the non-ABI group in the left DLPFC (i.e. CH13, and CH15). Altogether, the current findings were consistent between behavioral and hemodynamic findings, indicating that increased distractibility in ABI was possibly due to altered brain activity involving oxygen changes. The findings suggested that CIT1 and CIT2 can effectively demonstrate increases in auditory distractibility following ABI. A greater level of oxygen exchange in the left DLPFC is possibly a feature that underlies susceptibility to auditory distractibility and attention deficits following ABI. Furthermore, greater and, therefore, potentially abnormal oxygen exchanges in the region can serve as a biomarker to specifically detect auditory distractibility.

Our behavioral results showed that overall CIT performance was lower in patients with ABI than healthy adults. As the level of difficulty increased, CIT performance decreased. This trend was similar for both groups. The current findings are in line with the previous studies reporting worse performance in ABI than healthy control groups, and inadequate performances across various types of auditory attention in both patients with ABI and healthy controls^21,22,78–80^. Also, the findings were similar with our previous study^54^ reporting that younger and older adults showed a gradual decrease in performance accuracy across the different levels of CITs.

Interestingly, there existed a between-group difference. RCS scores between CIT1 and CIT2 were significantly different in both ABI and non-ABI group, while those between CIT2 and CIT3 were significant in the non-ABI group alone (Fig. 1). Considering that CIT1 and CIT2 required selective listening to target contours in the presence of distraction, our findings indicated that patients with ABI were susceptible to auditory distraction and their performance declined as the level of distraction increased (note that environmental sounds were used as distraction in CIT1, while target-like distracting sounds were used in CIT2). Our findings are supported by the concept of distractibility, which is considered key for leading to attention deficits in patients with ABI^15–17^. In actual fact, the declined performance in CIT1 and CIT2 (selective attention against auditory distraction) were similar to previous studies that compared and reported deficits in selective auditory attention in patients with ABI as compared to healthy controls^21,22,78–80^. An insignificant difference between CIT2 and CIT3 can be interpreted as a cognitive threshold existing in patients with ABI. Since CIT3 requires an ability to selectively attend to target contour presented by a specific instrument timbre and to shift the focus from one to another according to the visual cue given on the screen, it seems that this task involves higher-order cognition (i.e., mental flexibility) than that required in CIT2. The current findings were similar with our previous study^54^ that showed CIT2 is sensitive enough to detect a cognitive decline existing in elderly group compared to young adult group.

Our vector plane analysis showed that the non-ABI group fell into phases 1 to 2, while those in the ABI group mostly fell into phases 4 to 5 (see Fig. 2), which indicated that greater and, thus, potentially abnormal oxygen exchanges were required in the ABI group. More importantly, PRI analysis confirmed the trend statistically; the PRIs increased significantly more in the ABI group than in the non-ABI group in the left DLPFC (see Fig. 2, CH13 and CH15) while they performed the CIT1 and CIT2 (selective target contour identification against different types of auditory distraction). This result appeared to be due to the following: 1) increases in angle *k*, which meant ∆COE with decreased ∆CBV and 2) a longer scalar L, which was mainly attributed to total amount of changes in ∆COE and ∆CBV. That is, the greater oxygen exchanges in the patients with ABI as indicated by the hypoxia/ischemia phase was reflected both by high oxygen consumption (∆HHb > ∆HbO_2_) and a lack of sufficient oxygen supply (∆CBV < 0).

Because no studies have examined oxygen exchange (using PRI analysis) in patients with ABI in relation to their cognitive performance, we first compared our findings to a group of studies that examined cognitive loads and oxygen demands (i.e., actual and simulated driving situations) in healthy adults. Yoshino *et al*.^81^ found that oxygen metabolic demands varied with vehicle manipulation, that is to say, greater during deceleration as opposed to acceleration, and interpreted the demands that increased as an increase in the difficulty of a given task. Oka *et al*.^82^ reported a higher PRI value in the right than left premotor cortex during left curve driving, which indicated the occurrence of frequent oxygen exchanges possibly due to greater neural activities. Both studies implied that the driving tasks involving various contexts facilitated the usage of cognitive functions and changes in oxygen metabolism in the prefrontal regions. The previous findings are therefore in line with our findings that indicated that the cognitive demands associated with CITs lead to hemodynamic changes involving oxygen level exchange.

The current findings addressed previous mixed results reporting either hypo- or hyper-activation in oxygenated hemoglobin during cognitive task performance in patients with ABI^75,83–87^. Our findings in the HbO_2_-HHb pattern analyses confirmed the need for both indices in examining oxygen metabolism of patients with ABI (see Fig. 2 for the different activation patterns between HbO_2_ and HHb, which are distinctive between groups). Additional attempts to include oxygen-related multiple indices revealed the altered hemodynamic responses are ABI-specific (hypoxia and ischemia as indicated by HHb > HbO_2_ and CBV < 0)^55,56^.

Moreover, stronger oxygen metabolic demands observed in the ABI group can be interpreted as an impairment of cerebral oxygen exchange that acts as an underlying mechanism of susceptibility to auditory distraction and auditory attention deficits in patients with ABI. That is, insufficient oxygen supply due to dysfunctions in neuronal activation and connectivity caused high oxygen demands (i.e., ΔHHb is greater than ΔHbO_2_. These results are similar with previous findings reporting that healthy adults showed regional increases in HbO_2_ accompanied by HHb decreases. However, patients with cerebral ischemia or brain tumors have been reported to exhibit HHb increases together with HbO_2_ increases^88–91^. Since increasing tissue oxygenation is considered crucial in improving cognitive function for patients with ABI^26,92–94^ and TBI^95–97^, dysfunctions in oxygen metabolism can therefore underlie auditory cognitive impairment following ABI.

**Figure 4:**
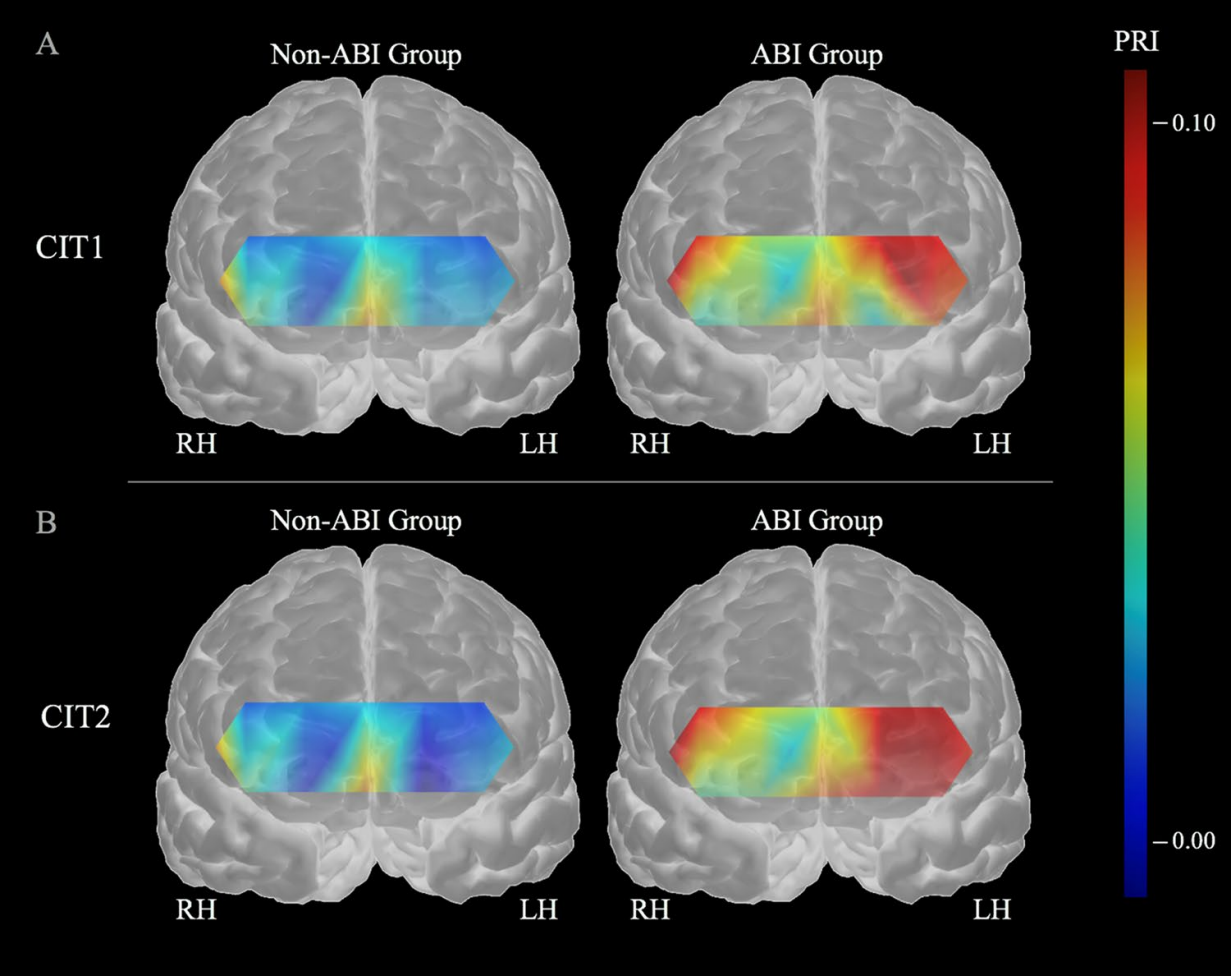
Differences in oxygen metabolic demands between groups during (**A**) CIT1 and (**B**) CIT2.

Our findings showed that the oxygen level exchange during CIT1 and CIT2 were prominent in the ABI group than in the non-ABI group in the left DLPFC (see Fig. 2, CHs 13 and 15 for PRI). This indicated that the left DLPFC was more sensitive to detect the oxygen metabolism-associated auditory dysfunction in the ABI group and, thus, can be used as a biomarker of auditory distractibility in ABI. Figure 4 shows stronger oxygen metabolic demands in the ABI than the non-ABI group and in the left than the right DLPFC. In general, the DLPFC controls higher-cognitive functions, such as working memory and executive control^98–100^. This region, also known as Brodmann area 46 (CH15 in the fNIRS data), receives information from the auditory cortex, and the stronger oxygen level exchange in the DLPFC is therefore likely to occur during the performance of a complex auditory task^101–104^, such as CIT1 and CIT2 that were used in our study.

In our behavioral findings, CIT2 performance significantly worsened compared with that of CIT1 indicating imposition of more cognitive loads. Although we did not find statistical significance, Fig. 4 clearly shows greater level of oxygen exchange in the left DLPFC in CIT2 than CIT1. This significant difference observed in the left DLPFC can be task specific as Jeong and Ryu (2016)^105^ reported prefrontal asymmetry in a timbre working memory task. Timbre is generally known as typically processed in the right hemisphere; however, when a timbre task is combined with a complex cognitive task, the left DLPFC can be dominantly activated. Timbre is one of the key factors in auditory scene analysis because it provides a perceptual clue of how one organizes perceived auditory surroundings^106,107^. When two people speak simultaneously, timbre cues aid in selectively attending to target information and higher-order cognitive processes, such as executive functioning that underlies goal-directed behavior, which is necessary in auditory scene analysis in concert with timbre discrimination^108^. In this study, we employed target melody detection utilizing timbre difference as a perceptual cue to differentiate a target during distraction. Unsurprisingly, the left DLPFC activation indicated the involvement of higher-order cognitive processing given by spatial timbre cues.

## Conclusion

The present study aimed to examine cerebral hemodynamic changes in individuals with ABI during melodic contour identification task. We utilized fNIRS to monitor the hemodynamic activity in the prefrontal cortex and analysed the HbO_2_ and HHb related indices with a vector-based analysis method. Our behavioral findings revealed significant differences between the ABI and non-ABI groups in CIT1 and CIT2 (target contour identification against environmental sounds and target-like distraction). The level of oxygen exchange in the left DLPFC during CIT1 and CIT2 increased significantly in the patients with ABI. These findings together indicated that CIT is able to discern auditory distractibility in patients with ABI. Furthermore, more frequent oxygen exchange in the left DLPFC is a possible underlying mechanism that leads to auditory attention deficits in these patients. Our results suggest that oxygen metabolic demands during melodic CITs can provide a surrogate biomarker of auditory dysfunction in patients with ABI as a result of stroke or TBI.

The novel attempts of the current study mostly rested on the use of fNIRS combined with the vector-based analysis, which enabled to evaluate cerebral oxygen metabolism with an efficient index. This method provided a clear way to compare interrelationships among diverse indices, including ∆HbO_2_, ∆HHb, ΔCBV and ΔCOE on the same vector plane and to classify abnormalities in oxygen metabolism using the definitions of each of the eight phases^81,82^. We, however, recognize some limitations of this particular study. The fNIRS was devised to observe PFC activation alone, and therefore, connectivity with other deep brain regions or the possible influence of damaged brain areas has not been accounted for. We therefore cannot determine whether the cognitive deficits were solely associated with oxygen metabolism in the PFC. Instead, this study focused on cerebral activity involving oxygen level exchange during a non-verbal auditory assessment (note that musical non-verbal information processing is expected to be less knowledge- and culture-dependent), which could be more widely available for use.

An urgent future study would thus combine diffuse correlation spectroscopy or employ fMRI to examine a direct relationship between multiple hemodynamic indices of fNIRS combined with vector-based phase analysis and cerebral metabolic rate of oxygen (CMRO_2_). Future study can also consist of a scaled-up experiment that examines the relationship between music and cognitive function in real-world situations (e.g. simulated and scenario-based auditory assessments in augmented or virtual reality). Another type of future study could broaden the scope of applying CITs to more patients with cognitive impairments, including dementia and mild cognitive impairment, and compare the data with various types of neuroimaging data (e.g. fMRI) to specify the essence of music assessment.

## Methods

### Participants

This study was approved by the Institutional Review Board of Hanyang University (IRB No. HYUH 2013-08-017) and the National Rehabilitation Centre (IRB No. 2015-040). All participants have provided written informed consent in accordance with the Declaration of Helsinki. Thirty-seven participants (healthy adults = 22, patients with ABI = 15) participated in the study. The patients with ABI were voluntarily recruited from the National Rehabilitation Centre of South Korea. The patients were eligible if they showed cognitive impairment at least in one domain on a neuropsychological battery, the Seoul Neuropsychological Screening Battery II (SNSB-Second edition)^109^. Patients who had a less than three months of regular involvement in musical activities and/or professional training, who had a minimal ability to understand the spoken instruction, and who were without sensory impairments were eligible to participate in the study. The average age of the patients was 53.60 years (SD = 8.88), and an average of 19.44 months (SD = 33.28) had passed after their brain injuries. The patients’ average years of education were 14.47 years (SD = 2.28). The SI Appendix, Table 5 lists the characteristics of the patients.

The non-ABI group was voluntarily recruited by advertising on the electronic board of Hanyang University. The average age of the control group was 55.77 years (SD = 5.98), and the patients’ average years of education were 12.77 years (SD = 3.40). The control group did not have a neurological medical history and present with no sensory impairments. The groups did not differ significantly for either age or education level [*t*_(35)_ = 0.8655, non-significant; *t*_(35)_ = −1.644, non-significant, respectively]. All participants were right-handed according to the Edinburgh Handedness Inventory^110^, and no participants were regularly involved with musical activities and/or professional training.

### Musical Stimuli and Task

Melodic contour identification task (CIT)^54^ was used in the present study. CIT was designed to measure the three types of attention: focused, selective, and alternating attention using melodic contours, including ascending, stationary, and descending. In CIT1, participants were presented with target melodic contours against environmental sounds and asked to identify the direction of contours. In CIT2, participants were presented with target melodic contours against target-like distractors (i.e., another melodic contour played by different instrument timbres) and were asked to identify a target contour presented in a predetermined instrument timbre. In CIT3, two melodic contours were presented, while participants were asked to shift their attention from one to another target contour and identify the direction of contours. For both CIT2 and CIT3, a visual cue (e.g., a picture of an instrument) was shown on the computer screen to inform about the instrument timbre of target contour.

For all CITs, melodic contours were randomly selected and presented in randomly selected instrument timbres. Two different melodic contours were simultaneously presented, and we took measures to avoid combinations being presented in the same two instrument timbres. The experiment took about 20 min to complete and behavioral and haemodynamic responses were recorded throughout the experiment. The contours were presented in one of these three synthesized instruments: piano, flute or string, and their amplitudes were identically normalized. All were generated using a musical instrument digital interface (MIDI) synthesizer that was connected to Logic Pro X, and the experimental apparatus was implemented on a computer using Visual Studio.

### fNIRS measurements

**Figure 5:**
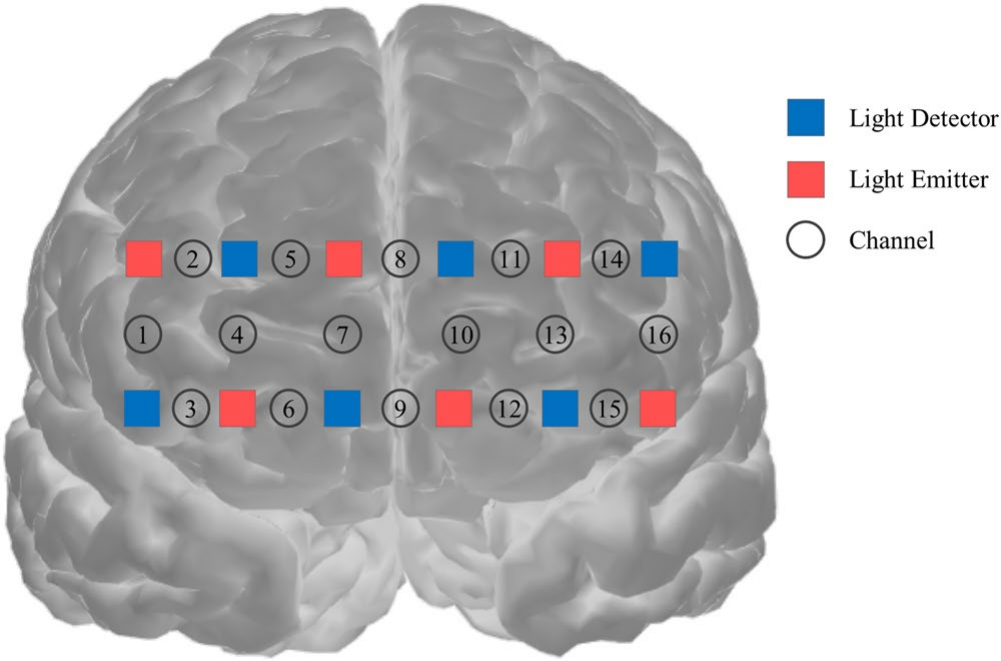
Location of the probe and channels. Red and blue boxes represent the locations of detector and emitter; circled numbers represent channel information. The center of the measurement unit was placed on the frontopolar region (Fpr) according to the international 10–20 system.

We used fNIRS (16-channel Spectratech OEG-16) to record cortical tissue oxygenation (HbO_2_) and deoxygenation (HHb) to measure the participant’s brain activity during the CITs^111–113^. The band-type fNIRS containing an array of 12 probes was attached to each participant’s forehead to obtain hemodynamic signals in the bilateral frontal cortex (Fig. 5). The probes were connected to the main board of the fNIRS, which communicated with a computer.

**Figure 6:**
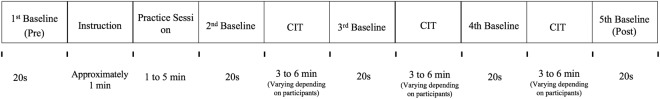
The flow of the experiment.

Participants had a short rest period and then the pre-stimulus baseline data were obtained for 20 seconds while they fixated their eyes on the centre of the monitor. A 20-second baseline was also obtained during inter-task rest periods and post-task period. Once the baseline data were obtained, the participants were presented with instruction and examples of melodic contours. The contours were delivered via a headphone with controlled volume, while the visual cues specifying the target musical instrument were presented to the participants on a monitor. The participants underwent a practice session to become familiar with the direction identification task. When their accuracy was over 80%, the main experimental session was administered. A total of 18 test items were presented in each CIT (a blocked design) and the order of CIT was randomized across participants (Fig. 6). The experiment was performed in a sound-proof room, in which light and temperature were controlled.

### Vector-based phase analysis for fNIRS data

The hemodynamic changes were obtained with a sampling rate of 0.65 s, and the data were converted to concentrated changes of hemoglobin using the modified Beer–Lambert law. Then, a zero-phase low- and high-pass filter with a cut-off frequency of 0.01 to 0.09 Hz was applied using matrix laboratory (MATLAB)^114–116^. The raw fNIRS data were collected and converted to concentrated changes of hemoglobin using the modified Beer–Lambert law^117^.

In this study, we employed a vector-based phase analysis to examine multifaceted aspects of cerebral hemodynamic changes^118–121^. The vector-based phase analysis devised by Kato is a method based on an orthogonal vector coordinate plane defined by four indices: ΔHbO_2_, ΔHHb, ΔCBV, and ΔCOE^121–124^. They are vector components of oxygenated hemoglobin, deoxygenated hemoglobin, total hemoglobin concentration, and oxygen level change in blood vessels, respectively^82,121,125^. ΔCBV and ΔCOE are obtained by rotating the vector coordinate plane defined by the ΔHbO_2_ and Δ HHb by 45° counterclockwise. The equations were like the following:

1 $${\rm{\Delta }}\mathrm{CBV}=\frac{{({\rm{\Delta }}\mathrm{HHb}+{\rm{\Delta }}\mathrm{HbO}}_{2})}{\sqrt{2}}$$

2 $${\rm{\Delta }}\mathrm{COE}=\frac{({\rm{\Delta }}\mathrm{HHb}\,-{{\rm{\Delta }}\mathrm{HbO}}_{2})\,}{\sqrt{2}}$$

The four indices are subject to calculate angle *k*, which represents the ratio of ∆COE to ∆CBV. Since a positive ΔCOE value (HHb > HbO_2_) indicates the hypoxic change due to greater consumption of oxygen resulting in increases of dioxygen in blood vessels, angle *k* indicates the degree of oxygen exchange^81,82,121,125^. Angle *k* can be obtained by the following equation (3).

3 $$k=Arc\,tan\,(\begin{array}{c}{\rm{\Delta }}\,{\rm{COE}}\\ {\rm{\Delta }}\,{\rm{CBV}}\end{array})+{45}^{\circ }(\,-\,{135}^{\circ }\le {\rm{k}}\le {225}^{\circ })$$

ΔCBV determines the hyperemic/ischemic state (if CBV is above zero, it is ischemia; if CBV is below zero, it is hyperemia) and ΔCOE determines the hyperoxic/hypoxic state (if COE is above zero, it is hypoxia; if COE is below zero, it is hyperoxia), together leading to changes in *k* value^82,123,126^. Hyperoxia is an increase in oxygen supply, which then increases the chances that the brain can utilize delivered oxygen and which can improve CMRO_2_. Hypoxia, in contrast, refers to the deprivation of an adequate oxygen supply in the brain tissues. Hyperemia is the increase in blood volume to the brain that meet the oxygen demands in the blood vessels, while ischemia means the decrease in the volume^127–129^.

**Figure 7:**
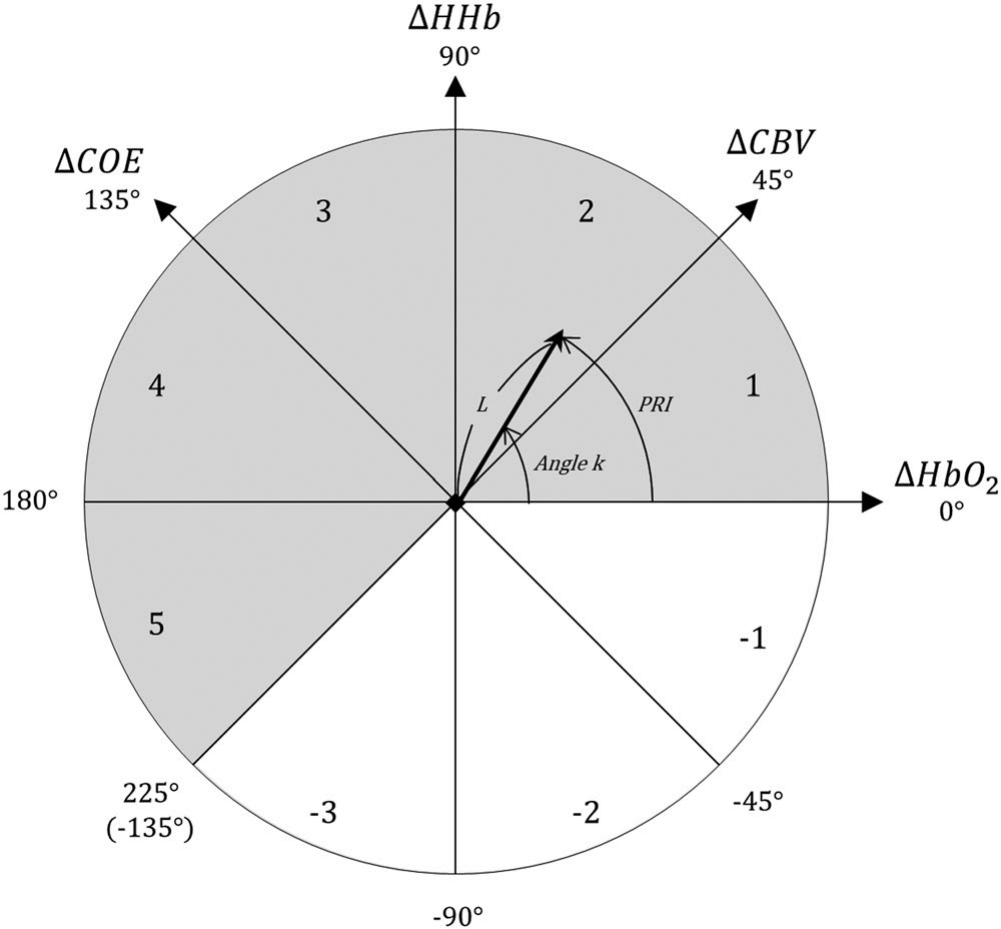
Polar coordinate plane of the vector-based phase.

Angle *k*, thus, can provide additional topological information concerning the various degrees of oxygen exchange (Fig. 7). Increases in *k* (from 0° to 225°) fall in phases 1 to 5, which reflect the increased hemodynamic activity involved in oxygen level exchange, while decreases in *k* (from −135° to 0°) fall into phases −1 to −3, which indicate no increases or weak changes (Please see SI Appendix, Table 6 for more details).

A scalar L, the length of the vector, shows the amplitude of the vector and indicate the amounts of hemoglobin variation^123^. Further, the four indices (∆COE, ∆CBV, *k* and *L*) were used to calculate a phase-associated response intensity (PRI), where *k*^*rad*^ refers to the degree converted to radians. The PRI denotes the intensity of hemodynamic responses involved in oxygen exchange^82^. Because the four indices are visualized in the same plane, their relationships as well as the changes across time are observable and readily interpretable.

4 $$|{\rm{L}}|=\sqrt{{{\rm{\Delta }}\mathrm{COE}}^{2}+{{\rm{\Delta }}\mathrm{CBV}}^{2}}$$

5 $${\rm{PRI}}={k}^{rad}\cdot L$$

### Statistical analysis

For the behavioral analysis, a two-way mixed analysis of variance (ANOVA) was performed. The independent variables were Group (ABI and non-ABI) and Task (CIT1, CIT2 and CIT3), and the dependent variable was the rate-corrected score (RCS), or the number of correct responses per second of activity. The RCS was estimated using c/ΣRT where c refers to the correct responses and RT refers to the response time^130,131^. For the hemodynamic analysis, a two-way mixed ANOVA was performed. The independent variables were Group (ABI and non-ABI) and task (pre-task, on-task and post-task, Note that the on-task included CIT1 and CIT2). The dependent variable was the PRIs from the vector-based phase analysis. Specifically, we focused on the hemodynamic data obtained from CHs 1, 2, 3, 13, 14 and 15 [CHs 1, 2 and 3 measured activation in the right dorsolateral prefrontal cortex (DLPFC), while CHs 13, 14 and 15 measured activation in the left DLPFC). These regions are sensitive to cognitive load^113,132^. All statistical analyses were performed using R.

## Electronic supplementary material

Supplementary Information

## Data Availability

The datasets generated during and/or analysed during the current study are available from the corresponding author on reasonable request.
