# Supplementary material for: High Oxygen Exchange to Music Indicates Auditory Distractibility in Acquired Brain Injury: An fNIRS Study with a Vector-Based Phase Analysis
Source: Sci Rep. 2018 Nov 13;8:16737. doi: 10.1038/s41598-018-35172-2 (PMC6233191; doi:10.1038/s41598-018-35172-2)
Supplement: Supplementary file 1 — Supplementary Information [file 41598_2018_35172_MOESM1_ESM.docx]

Supplementary Information for

**High Oxygen Exchange to Music Indicates Auditory Distractibility in Acquired Brain Injury: An fNIRS Study with a Vector-Based Phase Analysis**

Eunju Jeong^1,2*^, Ph.D.

Hokyoung Ryu^1,3^, Ph.D.

Joon-Ho Shin^4^, M.D.

Gyu Hyun Kwon^1,3^, Ph.D.

Geonsang Jo^1^

Ji-Yeong Lee^4^

Correspondence concerning this article should be addressed to Eunju Jeong. Email: ejeong@hanyang.ac.kr

**This file includes:** Tables 1 to 6

**Table 1. Descriptive statistics of HbO_2_ in each of the six channels**

| Task | | CH | ∆HbO_2_ | | | |  | ∆HHb | | | | | | |
| --- | --- | --- | --- | --- | --- | --- | --- | --- | --- | --- | --- | --- | --- | --- |
|  |  |  | Non-ABI (N = 22) | | ABI (N = 15) | |  | Non-ABI (N = 22) | | ABI (N = 15) | | |  |  |
|  |  |  | Mean | SD | Mean | SD |  | Mean | SD | | Mean | SD | |  |
| Pre | | 1 | 0.019 | 0.050 | 0.009 | 0.061 |  | 0.008 | 0.043 | | 0.004 | 0.025 | |  |
|  |  | 2 | -0.003 | 0.026 | 0.016 | 0.052 |  | 0.002 | 0.025 | | 0.001 | 0.034 | |  |
|  |  | 3 | 0.006 | 0.046 | -0.004 | 0.024 |  | 0.004 | 0.023 | | -0.009 | 0.017 | |  |
|  |  | 13 | -0.008 | 0.027 | 0.020 | 0.043 |  | 0.003 | 0.027 | | 0.002 | 0.036 | |  |
|  |  | 14 | -0.001 | 0.017 | 0.023 | 0.036 |  | 0.002 | 0.027 | | 0.000 | 0.031 | |  |
|  |  | 15 | 0.000 | 0.027 | 0.019 | 0.035 |  | 0.004 | 0.023 | | 0.006 | 0.024 | |  |
|  |  | Mean | 0.002 | 0.032 | 0.014 | 0.042 |  | 0.004 | 0.028 | | 0.001 | 0.028 | |  |
| CIT1 | | 1 | 0.000 | 0.003 | -0.001 | 0.002 |  | 0.001 | 0.002 | | 0.000 | 0.003 | |  |
|  |  | 2 | 0.000 | 0.002 | 0.001 | 0.002 |  | 0.001 | 0.003 | | 0.000 | 0.003 | |  |
|  |  | 3 | -0.001 | 0.003 | 0.000 | 0.003 |  | 0.001 | 0.002 | | 0.000 | 0.003 | |  |
|  |  | 13 | -0.002 | 0.003 | 0.001 | 0.004 |  | 0.001 | 0.003 | | 0.000 | 0.002 | |  |
|  |  | 14 | 0.000 | 0.003 | 0.000 | 0.002 |  | 0.002 | 0.003 | | 0.000 | 0.002 | |  |
|  |  | 15 | -0.001 | 0.004 | 0.000 | 0.002 |  | 0.001 | 0.002 | | 0.000 | 0.002 | |  |
|  |  | Mean | -0.001 | 0.003 | 0.000 | 0.002 |  | 0.001 | 0.002 | | 0.000 | 0.002 | |  |
| CIT2 | | 1 | -0.001 | 0.006 | -0.004 | 0.012 |  | -0.002 | 0.006 | | -0.001 | 0.006 | |  |
|  |  | 2 | 0.000 | 0.005 | -0.002 | 0.005 |  | -0.001 | 0.005 | | 0.000 | 0.005 | |  |
|  |  | 3 | -0.002 | 0.004 | -0.002 | 0.004 |  | -0.003 | 0.006 | | -0.001 | 0.005 | |  |
|  |  | 13 | -0.001 | 0.004 | 0.001 | 0.006 |  | -0.001 | 0.005 | | 0.001 | 0.005 | |  |
|  |  | 14 | 0.001 | 0.005 | 0.000 | 0.005 |  | -0.001 | 0.004 | | 0.000 | 0.004 | |  |
|  |  | 15 | -0.002 | 0.006 | -0.001 | 0.005 |  | -0.001 | 0.006 | | -0.002 | 0.004 | |  |
|  |  | Mean | -0.001 | 0.005 | -0.001 | 0.006 |  | -0.001 | 0.005 | | 0.000 | 0.005 | |  |
| Post | | 1 | 0.019 | 0.050 | 0.009 | 0.061 |  | 0.008 | 0.043 | | 0.004 | 0.025 | |  |
|  |  | 2 | -0.003 | 0.026 | 0.016 | 0.052 |  | 0.002 | 0.025 | | 0.001 | 0.034 | |  |
|  |  | 3 | 0.006 | 0.046 | -0.004 | 0.024 |  | 0.004 | 0.023 | | -0.009 | 0.017 | |  |
|  |  | 13 | -0.008 | 0.027 | 0.020 | 0.043 |  | 0.003 | 0.027 | | 0.002 | 0.036 | |  |
|  |  | 14 | -0.001 | 0.017 | 0.023 | 0.036 |  | 0.002 | 0.027 | | 0.000 | 0.031 | |  |
|  |  | 15 | 0.000 | 0.027 | 0.019 | 0.035 |  | 0.004 | 0.023 | | 0.006 | 0.024 | |  |
|  |  | Mean | 0.002 | 0.032 | 0.014 | 0.042 |  | 0.004 | 0.028 | | 0.001 | 0.028 | |  |

∆HHb: changes in deoxygenated haemoglobin, ∆HbO_2_: changes in oxygenated haemoglobin

**Table 2. Changes of HbO_2_ and HHb for Sample Data Set**

| Group  Time  (0.65s) | | HbO_2_ | | | | | | | | | | | | HHb | | | | | | | | | | | |
| --- | --- | --- | --- | --- | --- | --- | --- | --- | --- | --- | --- | --- | --- | --- | --- | --- | --- | --- | --- | --- | --- | --- | --- | --- | --- |
|  |  | CH1 | | CH2 | | CH3 | | CH13 | | CH14 | | CH15 | | CH1 | | CH2 | | CH3 | | CH13 | | CH14 | | CH15 | |
|  |  | M | SE | M | SE | M | SE | M | SE | M | SE | M | SE | M | SE | M | SE | M | SE | M | SE | M | SE | M | SE |
| Non-ABI | 1 | -0.0001 | 0.0095 | 0.0185 | 0.0110 | -0.0092 | 0.0115 | -0.0001 | 0.0095 | 0.0185 | 0.0110 | -0.0092 | 0.0115 | -0.0024 | 0.0160 | 0.0075 | 0.0145 | -0.0062 | 0.0132 | -0.0024 | 0.0160 | 0.0075 | 0.0145 | -0.0062 | 0.0132 |
|  | 2 | -0.0016 | 0.0095 | 0.0168 | 0.0103 | -0.0098 | 0.0115 | -0.0016 | 0.0095 | 0.0168 | 0.0103 | -0.0098 | 0.0115 | -0.0030 | 0.0158 | 0.0061 | 0.0140 | -0.0076 | 0.0131 | -0.0030 | 0.0158 | 0.0061 | 0.0140 | -0.0076 | 0.0131 |
|  | 3 | -0.0040 | 0.0098 | 0.0143 | 0.0098 | -0.0115 | 0.0114 | -0.0040 | 0.0098 | 0.0143 | 0.0098 | -0.0115 | 0.0114 | -0.0036 | 0.0156 | 0.0049 | 0.0135 | -0.0090 | 0.0128 | -0.0036 | 0.0156 | 0.0049 | 0.0135 | -0.0090 | 0.0128 |
|  | 4 | -0.0071 | 0.0105 | 0.0112 | 0.0094 | -0.0140 | 0.0112 | -0.0071 | 0.0105 | 0.0112 | 0.0094 | -0.0140 | 0.0112 | -0.0041 | 0.0154 | 0.0038 | 0.0130 | -0.0102 | 0.0123 | -0.0041 | 0.0154 | 0.0038 | 0.0130 | -0.0102 | 0.0123 |
|  | 5 | -0.0108 | 0.0117 | 0.0078 | 0.0093 | -0.0172 | 0.0110 | -0.0108 | 0.0117 | 0.0078 | 0.0093 | -0.0172 | 0.0110 | -0.0048 | 0.0152 | 0.0027 | 0.0126 | -0.0113 | 0.0117 | -0.0048 | 0.0152 | 0.0027 | 0.0126 | -0.0113 | 0.0117 |
|  | 6 | -0.0147 | 0.0133 | 0.0043 | 0.0094 | -0.0208 | 0.0107 | -0.0147 | 0.0133 | 0.0043 | 0.0094 | -0.0208 | 0.0107 | -0.0055 | 0.0152 | 0.0017 | 0.0122 | -0.0122 | 0.0111 | -0.0055 | 0.0152 | 0.0017 | 0.0122 | -0.0122 | 0.0111 |
|  | 7 | -0.0185 | 0.0150 | 0.0010 | 0.0097 | -0.0244 | 0.0104 | -0.0185 | 0.0150 | 0.0010 | 0.0097 | -0.0244 | 0.0104 | -0.0064 | 0.0152 | 0.0008 | 0.0118 | -0.0130 | 0.0105 | -0.0064 | 0.0152 | 0.0008 | 0.0118 | -0.0130 | 0.0105 |
|  | 8 | -0.0220 | 0.0165 | -0.0018 | 0.0101 | -0.0276 | 0.0102 | -0.0220 | 0.0165 | -0.0018 | 0.0101 | -0.0276 | 0.0102 | -0.0074 | 0.0152 | 0.0000 | 0.0115 | -0.0135 | 0.0101 | -0.0074 | 0.0152 | 0.0000 | 0.0115 | -0.0135 | 0.0101 |
|  | 9 | -0.0248 | 0.0178 | -0.0039 | 0.0105 | -0.0300 | 0.0101 | -0.0248 | 0.0178 | -0.0039 | 0.0105 | -0.0300 | 0.0101 | -0.0086 | 0.0152 | -0.0009 | 0.0112 | -0.0139 | 0.0099 | -0.0086 | 0.0152 | -0.0009 | 0.0112 | -0.0139 | 0.0099 |
|  | 10 | -0.0267 | 0.0187 | -0.0051 | 0.0109 | -0.0314 | 0.0101 | -0.0267 | 0.0187 | -0.0051 | 0.0109 | -0.0314 | 0.0101 | -0.0097 | 0.0152 | -0.0018 | 0.0110 | -0.0141 | 0.0100 | -0.0097 | 0.0152 | -0.0018 | 0.0110 | -0.0141 | 0.0100 |
|  | 11 | -0.0276 | 0.0191 | -0.0054 | 0.0112 | -0.0316 | 0.0101 | -0.0276 | 0.0191 | -0.0054 | 0.0112 | -0.0316 | 0.0101 | -0.0110 | 0.0152 | -0.0028 | 0.0108 | -0.0143 | 0.0104 | -0.0110 | 0.0152 | -0.0028 | 0.0108 | -0.0143 | 0.0104 |
|  | 12 | -0.0275 | 0.0190 | -0.0048 | 0.0115 | -0.0305 | 0.0102 | -0.0275 | 0.0190 | -0.0048 | 0.0115 | -0.0305 | 0.0102 | -0.0124 | 0.0152 | -0.0041 | 0.0107 | -0.0144 | 0.0110 | -0.0124 | 0.0152 | -0.0041 | 0.0107 | -0.0144 | 0.0110 |
|  | 13 | -0.0267 | 0.0187 | -0.0034 | 0.0117 | -0.0287 | 0.0102 | -0.0267 | 0.0187 | -0.0034 | 0.0117 | -0.0287 | 0.0102 | -0.0140 | 0.0152 | -0.0055 | 0.0107 | -0.0149 | 0.0115 | -0.0140 | 0.0152 | -0.0055 | 0.0107 | -0.0149 | 0.0115 |
|  | 14 | -0.0254 | 0.0182 | -0.0048 | 0.0117 | -0.0286 | 0.0105 | -0.0254 | 0.0182 | -0.0048 | 0.0117 | -0.0286 | 0.0105 | -0.0150 | 0.0153 | -0.0067 | 0.0108 | -0.0147 | 0.0121 | -0.0150 | 0.0153 | -0.0067 | 0.0108 | -0.0147 | 0.0121 |
|  | 15 | -0.0239 | 0.0175 | 0.0016 | 0.0119 | -0.0213 | 0.0104 | -0.0239 | 0.0175 | 0.0016 | 0.0119 | -0.0213 | 0.0104 | -0.0158 | 0.0154 | -0.0087 | 0.0110 | -0.0153 | 0.0125 | -0.0158 | 0.0154 | -0.0087 | 0.0110 | -0.0153 | 0.0125 |
|  | 16 | -0.0212 | 0.0170 | 0.0038 | 0.0123 | -0.0171 | 0.0106 | -0.0212 | 0.0170 | 0.0038 | 0.0123 | -0.0171 | 0.0106 | -0.0187 | 0.0156 | -0.0118 | 0.0113 | -0.0170 | 0.0130 | -0.0187 | 0.0156 | -0.0118 | 0.0113 | -0.0170 | 0.0130 |
|  | 17 | -0.0182 | 0.0165 | 0.0064 | 0.0126 | -0.0090 | 0.0104 | -0.0182 | 0.0165 | 0.0064 | 0.0126 | -0.0090 | 0.0104 | -0.0254 | 0.0156 | -0.0171 | 0.0114 | -0.0210 | 0.0137 | -0.0254 | 0.0156 | -0.0171 | 0.0114 | -0.0210 | 0.0137 |
|  | 18 | -0.0157 | 0.0165 | 0.0071 | 0.0125 | -0.0063 | 0.0113 | -0.0157 | 0.0165 | 0.0071 | 0.0125 | -0.0063 | 0.0113 | -0.0217 | 0.0145 | -0.0167 | 0.0116 | -0.0209 | 0.0128 | -0.0217 | 0.0145 | -0.0167 | 0.0116 | -0.0209 | 0.0128 |
|  | 19 | -0.0147 | 0.0165 | 0.0122 | 0.0137 | -0.0032 | 0.0114 | -0.0147 | 0.0165 | 0.0122 | 0.0137 | -0.0032 | 0.0114 | -0.0277 | 0.0160 | -0.0219 | 0.0128 | -0.0246 | 0.0145 | -0.0277 | 0.0160 | -0.0219 | 0.0128 | -0.0246 | 0.0145 |
|  | 20 | -0.0137 | 0.0169 | 0.0144 | 0.0142 | 0.0006 | 0.0117 | -0.0137 | 0.0169 | 0.0144 | 0.0142 | 0.0006 | 0.0117 | -0.0304 | 0.0160 | -0.0247 | 0.0132 | -0.0270 | 0.0151 | -0.0304 | 0.0160 | -0.0247 | 0.0132 | -0.0270 | 0.0151 |
|  | 21 | -0.0135 | 0.0174 | 0.0160 | 0.0145 | 0.0036 | 0.0120 | -0.0135 | 0.0174 | 0.0160 | 0.0145 | 0.0036 | 0.0120 | -0.0327 | 0.0158 | -0.0269 | 0.0136 | -0.0290 | 0.0157 | -0.0327 | 0.0158 | -0.0269 | 0.0136 | -0.0290 | 0.0157 |
|  | 22 | -0.0144 | 0.0180 | 0.0166 | 0.0146 | 0.0057 | 0.0124 | -0.0144 | 0.0180 | 0.0166 | 0.0146 | 0.0057 | 0.0124 | -0.0342 | 0.0155 | -0.0281 | 0.0139 | -0.0302 | 0.0163 | -0.0342 | 0.0155 | -0.0281 | 0.0139 | -0.0302 | 0.0163 |
|  | 23 | -0.0162 | 0.0185 | 0.0163 | 0.0145 | 0.0070 | 0.0129 | -0.0162 | 0.0185 | 0.0163 | 0.0145 | 0.0070 | 0.0129 | -0.0348 | 0.0150 | -0.0282 | 0.0141 | -0.0301 | 0.0167 | -0.0348 | 0.0150 | -0.0282 | 0.0141 | -0.0301 | 0.0167 |
|  | 24 | -0.0188 | 0.0190 | 0.0149 | 0.0141 | 0.0075 | 0.0137 | -0.0188 | 0.0190 | 0.0149 | 0.0141 | 0.0075 | 0.0137 | -0.0341 | 0.0144 | -0.0269 | 0.0140 | -0.0286 | 0.0172 | -0.0341 | 0.0144 | -0.0269 | 0.0140 | -0.0286 | 0.0172 |
|  | 25 | -0.0220 | 0.0194 | 0.0126 | 0.0136 | 0.0074 | 0.0146 | -0.0220 | 0.0194 | 0.0126 | 0.0136 | 0.0074 | 0.0146 | -0.0320 | 0.0138 | -0.0243 | 0.0137 | -0.0255 | 0.0175 | -0.0320 | 0.0138 | -0.0243 | 0.0137 | -0.0255 | 0.0175 |
|  | 26 | -0.0256 | 0.0198 | 0.0095 | 0.0129 | 0.0067 | 0.0157 | -0.0256 | 0.0198 | 0.0095 | 0.0129 | 0.0067 | 0.0157 | -0.0285 | 0.0131 | -0.0204 | 0.0132 | -0.0208 | 0.0178 | -0.0285 | 0.0131 | -0.0204 | 0.0132 | -0.0208 | 0.0178 |
|  | 27 | -0.0286 | 0.0201 | 0.0062 | 0.0123 | 0.0059 | 0.0167 | -0.0286 | 0.0201 | 0.0062 | 0.0123 | 0.0059 | 0.0167 | -0.0239 | 0.0125 | -0.0159 | 0.0125 | -0.0152 | 0.0179 | -0.0239 | 0.0125 | -0.0159 | 0.0125 | -0.0152 | 0.0179 |
|  | 28 | -0.0322 | 0.0204 | 0.0021 | 0.0118 | 0.0042 | 0.0178 | -0.0322 | 0.0204 | 0.0021 | 0.0118 | 0.0042 | 0.0178 | -0.0178 | 0.0121 | -0.0098 | 0.0116 | -0.0078 | 0.0181 | -0.0178 | 0.0121 | -0.0098 | 0.0116 | -0.0078 | 0.0181 |
|  | 29 | -0.0348 | 0.0205 | -0.0018 | 0.0113 | 0.0023 | 0.0187 | -0.0348 | 0.0205 | -0.0018 | 0.0113 | 0.0023 | 0.0187 | -0.0111 | 0.0118 | -0.0037 | 0.0105 | -0.0004 | 0.0180 | -0.0111 | 0.0118 | -0.0037 | 0.0105 | -0.0004 | 0.0180 |
|  | 30 | -0.0366 | 0.0205 | -0.0052 | 0.0110 | 0.0002 | 0.0195 | -0.0366 | 0.0205 | -0.0052 | 0.0110 | 0.0002 | 0.0195 | -0.0043 | 0.0117 | 0.0022 | 0.0095 | 0.0068 | 0.0178 | -0.0043 | 0.0117 | 0.0022 | 0.0095 | 0.0068 | 0.0178 |
|  | 31 | -0.0397 | 0.0201 | -0.0099 | 0.0107 | -0.0054 | 0.0202 | -0.0397 | 0.0201 | -0.0099 | 0.0107 | -0.0054 | 0.0202 | 0.0040 | 0.0120 | 0.0102 | 0.0085 | 0.0151 | 0.0175 | 0.0040 | 0.0120 | 0.0102 | 0.0085 | 0.0151 | 0.0175 |
|  | 32 | -0.0376 | 0.0195 | -0.0108 | 0.0104 | -0.0051 | 0.0205 | -0.0376 | 0.0195 | -0.0108 | 0.0104 | -0.0051 | 0.0205 | 0.0083 | 0.0120 | 0.0124 | 0.0077 | 0.0186 | 0.0168 | 0.0083 | 0.0120 | 0.0124 | 0.0077 | 0.0186 | 0.0168 |
| ABI | 1 | -0.0206 | 0.0100 | -0.0241 | 0.0132 | -0.0197 | 0.0121 | -0.0206 | 0.0100 | -0.0241 | 0.0132 | -0.0197 | 0.0121 | -0.0004 | 0.0102 | 0.0009 | 0.0122 | 0.0042 | 0.0096 | -0.0004 | 0.0102 | 0.0009 | 0.0122 | 0.0042 | 0.0096 |
|  | 2 | -0.0212 | 0.0120 | -0.0244 | 0.0125 | -0.0195 | 0.0122 | -0.0212 | 0.0120 | -0.0244 | 0.0125 | -0.0195 | 0.0122 | -0.0011 | 0.0104 | 0.0001 | 0.0120 | 0.0027 | 0.0095 | -0.0011 | 0.0104 | 0.0001 | 0.0120 | 0.0027 | 0.0095 |
|  | 3 | -0.0212 | 0.0140 | -0.0242 | 0.0121 | -0.0188 | 0.0125 | -0.0212 | 0.0140 | -0.0242 | 0.0121 | -0.0188 | 0.0125 | -0.0017 | 0.0108 | -0.0007 | 0.0119 | 0.0014 | 0.0093 | -0.0017 | 0.0108 | -0.0007 | 0.0119 | 0.0014 | 0.0093 |
|  | 4 | -0.0208 | 0.0157 | -0.0236 | 0.0119 | -0.0176 | 0.0130 | -0.0208 | 0.0157 | -0.0236 | 0.0119 | -0.0176 | 0.0130 | -0.0025 | 0.0114 | -0.0015 | 0.0118 | 0.0004 | 0.0091 | -0.0025 | 0.0114 | -0.0015 | 0.0118 | 0.0004 | 0.0091 |
|  | 5 | -0.0203 | 0.0172 | -0.0227 | 0.0121 | -0.0161 | 0.0135 | -0.0203 | 0.0172 | -0.0227 | 0.0121 | -0.0161 | 0.0135 | -0.0034 | 0.0122 | -0.0023 | 0.0119 | -0.0005 | 0.0089 | -0.0034 | 0.0122 | -0.0023 | 0.0119 | -0.0005 | 0.0089 |
|  | 6 | -0.0201 | 0.0184 | -0.0217 | 0.0124 | -0.0143 | 0.0140 | -0.0201 | 0.0184 | -0.0217 | 0.0124 | -0.0143 | 0.0140 | -0.0048 | 0.0129 | -0.0032 | 0.0120 | -0.0012 | 0.0087 | -0.0048 | 0.0129 | -0.0032 | 0.0120 | -0.0012 | 0.0087 |
|  | 7 | -0.0202 | 0.0193 | -0.0208 | 0.0129 | -0.0125 | 0.0146 | -0.0202 | 0.0193 | -0.0208 | 0.0129 | -0.0125 | 0.0146 | -0.0065 | 0.0136 | -0.0044 | 0.0122 | -0.0018 | 0.0085 | -0.0065 | 0.0136 | -0.0044 | 0.0122 | -0.0018 | 0.0085 |
|  | 8 | -0.0209 | 0.0200 | -0.0201 | 0.0135 | -0.0107 | 0.0151 | -0.0209 | 0.0200 | -0.0201 | 0.0135 | -0.0107 | 0.0151 | -0.0086 | 0.0142 | -0.0057 | 0.0125 | -0.0022 | 0.0085 | -0.0086 | 0.0142 | -0.0057 | 0.0125 | -0.0022 | 0.0085 |
|  | 9 | -0.0220 | 0.0205 | -0.0197 | 0.0141 | -0.0089 | 0.0156 | -0.0220 | 0.0205 | -0.0197 | 0.0141 | -0.0089 | 0.0156 | -0.0109 | 0.0148 | -0.0072 | 0.0129 | -0.0024 | 0.0087 | -0.0109 | 0.0148 | -0.0072 | 0.0129 | -0.0024 | 0.0087 |
|  | 10 | -0.0234 | 0.0207 | -0.0198 | 0.0148 | -0.0073 | 0.0160 | -0.0234 | 0.0207 | -0.0198 | 0.0148 | -0.0073 | 0.0160 | -0.0132 | 0.0152 | -0.0088 | 0.0134 | -0.0024 | 0.0092 | -0.0132 | 0.0152 | -0.0088 | 0.0134 | -0.0024 | 0.0092 |
|  | 11 | -0.0248 | 0.0207 | -0.0202 | 0.0154 | -0.0058 | 0.0162 | -0.0248 | 0.0207 | -0.0202 | 0.0154 | -0.0058 | 0.0162 | -0.0152 | 0.0154 | -0.0104 | 0.0140 | -0.0021 | 0.0100 | -0.0152 | 0.0154 | -0.0104 | 0.0140 | -0.0021 | 0.0100 |
|  | 12 | -0.0258 | 0.0204 | -0.0210 | 0.0159 | -0.0045 | 0.0164 | -0.0258 | 0.0204 | -0.0210 | 0.0159 | -0.0045 | 0.0164 | -0.0167 | 0.0154 | -0.0118 | 0.0146 | -0.0015 | 0.0108 | -0.0167 | 0.0154 | -0.0118 | 0.0146 | -0.0015 | 0.0108 |
|  | 13 | -0.0261 | 0.0198 | -0.0220 | 0.0164 | -0.0033 | 0.0162 | -0.0261 | 0.0198 | -0.0220 | 0.0164 | -0.0033 | 0.0162 | -0.0174 | 0.0151 | -0.0129 | 0.0151 | -0.0006 | 0.0114 | -0.0174 | 0.0151 | -0.0129 | 0.0151 | -0.0006 | 0.0114 |
|  | 14 | -0.0256 | 0.0191 | -0.0230 | 0.0168 | -0.0021 | 0.0158 | -0.0256 | 0.0191 | -0.0230 | 0.0168 | -0.0021 | 0.0158 | -0.0173 | 0.0146 | -0.0135 | 0.0154 | 0.0007 | 0.0118 | -0.0173 | 0.0146 | -0.0135 | 0.0154 | 0.0007 | 0.0118 |
|  | 15 | -0.0260 | 0.0183 | -0.0239 | 0.0169 | 0.0014 | 0.0149 | -0.0260 | 0.0183 | -0.0239 | 0.0169 | 0.0014 | 0.0149 | -0.0174 | 0.0135 | -0.0133 | 0.0155 | 0.0024 | 0.0117 | -0.0174 | 0.0135 | -0.0133 | 0.0155 | 0.0024 | 0.0117 |
|  | 16 | -0.0228 | 0.0175 | -0.0250 | 0.0170 | 0.0002 | 0.0137 | -0.0228 | 0.0175 | -0.0250 | 0.0170 | 0.0002 | 0.0137 | -0.0149 | 0.0131 | -0.0133 | 0.0155 | 0.0039 | 0.0115 | -0.0149 | 0.0131 | -0.0133 | 0.0155 | 0.0039 | 0.0115 |
|  | 17 | -0.0195 | 0.0170 | -0.0251 | 0.0168 | 0.0016 | 0.0122 | -0.0195 | 0.0170 | -0.0251 | 0.0168 | 0.0016 | 0.0122 | -0.0125 | 0.0124 | -0.0123 | 0.0152 | 0.0058 | 0.0108 | -0.0125 | 0.0124 | -0.0123 | 0.0152 | 0.0058 | 0.0108 |
|  | 18 | -0.0151 | 0.0168 | -0.0242 | 0.0163 | 0.0032 | 0.0105 | -0.0151 | 0.0168 | -0.0242 | 0.0163 | 0.0032 | 0.0105 | -0.0104 | 0.0119 | -0.0107 | 0.0147 | 0.0080 | 0.0099 | -0.0104 | 0.0119 | -0.0107 | 0.0147 | 0.0080 | 0.0099 |
|  | 19 | -0.0108 | 0.0167 | -0.0227 | 0.0156 | 0.0055 | 0.0087 | -0.0108 | 0.0167 | -0.0227 | 0.0156 | 0.0055 | 0.0087 | -0.0063 | 0.0115 | -0.0083 | 0.0141 | 0.0100 | 0.0089 | -0.0063 | 0.0115 | -0.0083 | 0.0141 | 0.0100 | 0.0089 |
|  | 20 | -0.0068 | 0.0168 | -0.0205 | 0.0146 | 0.0080 | 0.0071 | -0.0068 | 0.0168 | -0.0205 | 0.0146 | 0.0080 | 0.0071 | -0.0035 | 0.0114 | -0.0059 | 0.0132 | 0.0120 | 0.0079 | -0.0035 | 0.0114 | -0.0059 | 0.0132 | 0.0120 | 0.0079 |
|  | 21 | 0.0017 | 0.0165 | -0.0166 | 0.0134 | 0.0101 | 0.0063 | 0.0017 | 0.0165 | -0.0166 | 0.0134 | 0.0101 | 0.0063 | 0.0008 | 0.0109 | -0.0033 | 0.0123 | 0.0131 | 0.0075 | 0.0008 | 0.0109 | -0.0033 | 0.0123 | 0.0131 | 0.0075 |
|  | 22 | -0.0009 | 0.0173 | -0.0141 | 0.0120 | 0.0142 | 0.0065 | -0.0009 | 0.0173 | -0.0141 | 0.0120 | 0.0142 | 0.0065 | -0.0002 | 0.0111 | -0.0006 | 0.0113 | 0.0160 | 0.0071 | -0.0002 | 0.0111 | -0.0006 | 0.0113 | 0.0160 | 0.0071 |
|  | 23 | 0.0004 | 0.0178 | -0.0105 | 0.0105 | 0.0177 | 0.0076 | 0.0004 | 0.0178 | -0.0105 | 0.0105 | 0.0177 | 0.0076 | 0.0001 | 0.0108 | 0.0017 | 0.0101 | 0.0175 | 0.0075 | 0.0001 | 0.0108 | 0.0017 | 0.0101 | 0.0175 | 0.0075 |
|  | 24 | -0.0016 | 0.0188 | -0.0087 | 0.0088 | 0.0205 | 0.0092 | -0.0016 | 0.0188 | -0.0087 | 0.0088 | 0.0205 | 0.0092 | -0.0019 | 0.0106 | 0.0025 | 0.0093 | 0.0176 | 0.0085 | -0.0019 | 0.0106 | 0.0025 | 0.0093 | 0.0176 | 0.0085 |
|  | 25 | -0.0025 | 0.0212 | -0.0066 | 0.0083 | 0.0216 | 0.0107 | -0.0025 | 0.0212 | -0.0066 | 0.0083 | 0.0216 | 0.0107 | -0.0033 | 0.0108 | 0.0046 | 0.0078 | 0.0174 | 0.0089 | -0.0033 | 0.0108 | 0.0046 | 0.0078 | 0.0174 | 0.0089 |
|  | 26 | -0.0035 | 0.0221 | -0.0017 | 0.0072 | 0.0260 | 0.0123 | -0.0035 | 0.0221 | -0.0017 | 0.0072 | 0.0260 | 0.0123 | -0.0041 | 0.0093 | 0.0058 | 0.0068 | 0.0191 | 0.0100 | -0.0041 | 0.0093 | 0.0058 | 0.0068 | 0.0191 | 0.0100 |
|  | 27 | -0.0035 | 0.0249 | -0.0009 | 0.0072 | 0.0267 | 0.0134 | -0.0035 | 0.0249 | -0.0009 | 0.0072 | 0.0267 | 0.0134 | -0.0054 | 0.0089 | 0.0054 | 0.0059 | 0.0181 | 0.0106 | -0.0054 | 0.0089 | 0.0054 | 0.0059 | 0.0181 | 0.0106 |
|  | 28 | -0.0156 | 0.0257 | 0.0002 | 0.0085 | 0.0231 | 0.0144 | -0.0156 | 0.0257 | 0.0002 | 0.0085 | 0.0231 | 0.0144 | -0.0096 | 0.0087 | 0.0038 | 0.0058 | 0.0159 | 0.0111 | -0.0096 | 0.0087 | 0.0038 | 0.0058 | 0.0159 | 0.0111 |
|  | 29 | -0.0103 | 0.0276 | -0.0147 | 0.0144 | 0.0127 | 0.0122 | -0.0103 | 0.0276 | -0.0147 | 0.0144 | 0.0127 | 0.0122 | -0.0137 | 0.0091 | -0.0065 | 0.0102 | 0.0049 | 0.0071 | -0.0137 | 0.0091 | -0.0065 | 0.0102 | 0.0049 | 0.0071 |
|  | 30 | -0.0186 | 0.0284 | -0.0027 | 0.0119 | 0.0247 | 0.0158 | -0.0186 | 0.0284 | -0.0027 | 0.0119 | 0.0247 | 0.0158 | -0.0132 | 0.0091 | 0.0031 | 0.0066 | 0.0136 | 0.0116 | -0.0132 | 0.0091 | 0.0031 | 0.0066 | 0.0136 | 0.0116 |
|  | 31 | -0.0235 | 0.0280 | -0.0045 | 0.0133 | 0.0220 | 0.0160 | -0.0235 | 0.0280 | -0.0045 | 0.0133 | 0.0220 | 0.0160 | -0.0158 | 0.0092 | 0.0011 | 0.0076 | 0.0105 | 0.0119 | -0.0158 | 0.0092 | 0.0011 | 0.0076 | 0.0105 | 0.0119 |
|  | 32 | -0.0279 | 0.0267 | -0.0067 | 0.0140 | 0.0184 | 0.0159 | -0.0279 | 0.0267 | -0.0067 | 0.0140 | 0.0184 | 0.0159 | -0.0179 | 0.0092 | -0.0010 | 0.0086 | 0.0069 | 0.0124 | -0.0179 | 0.0092 | -0.0010 | 0.0086 | 0.0069 | 0.0124 |

**Table 3. Mean and SD of Peak Time and HbO_2_ for Sample Data Set**

|  | Peak Time (sec) | | | |  | HbO_2_ | | | |
| --- | --- | --- | --- | --- | --- | --- | --- | --- | --- |
| CH | Non-ABI (N = 22) | | ABI (N = 15) | |  | Non-ABI (N = 22) | | ABI (N = 15) | |
|  | Mean | SD | Mean | SD |  | Mean | SD | Mean | SD |
| 1 | 13.5333 | 11.4696 | 12.0667 | 12.6122 |  | 0.0612 | 0.1575 | 0.0735 | 0.1997 |
| 2 | 15.2667 | 11.2407 | 16.4667 | 11.5935 |  | 0.0867 | 0.1057 | 0.0558 | 0.1618 |
| 3 | 12.4667 | 11.2368 | 16.8667 | 12.2058 |  | 0.0651 | 0.0981 | 0.1145 | 0.1317 |
| 13 | 23.0667 | 12.4526 | 19.4667 | 10.7495 |  | 0.0524 | 0.0656 | 0.1323 | 0.2670 |
| 14 | 17.4667 | 13.8299 | 22.4000 | 10.6757 |  | 0.0332 | 0.0492 | 0.0368 | 0.1026 |
| 15 | 22.3333 | 9.7736 | 13.8667 | 11.4447 |  | 0.0675 | 0.0804 | 0.0246 | 0.0625 |

**Table 4. Descriptive analysis of vectors in the six fNIRS channels**

| Task | CH | ∆COE | | | | | ∆CBV | | | | | L | | | | | k | | | | | PRI | | | |
| --- | --- | --- | --- | --- | --- | --- | --- | --- | --- | --- | --- | --- | --- | --- | --- | --- | --- | --- | --- | --- | --- | --- | --- | --- | --- |
|  |  | Non-ABI | | ABI | | Non-ABI | | | ABI | | Non-ABI | | | ABI | | Non-ABI | | | ABI | | Non-ABI | | | ABI | |
|  |  | Mean | SD | Mean | SD | Mean | | SD | Mean | SD | Mean | | SD | Mean | SD | Mean | | SD | Mean | SD | Mean | | SD | Mean | SD |
| Pre-CIT1 | 1 | 0.0084 | 0.0297 | 0.0039 | 0.0374 | -0.0190 | | 0.0581 | -0.0095 | 0.0562 | 0.0428 | | 0.0528 | 0.0506 | 0.0437 | -146.07 | | 79.95 | 146.85 | 104.54 | 0.0782 | | 0.2113 | 0.1072 | 0.1367 |
|  | 2 | -0.0022 | 0.0174 | 0.0101 | 0.0239 | 0.0018 | | 0.0334 | -0.0109 | 0.0586 | 0.0276 | | 0.0250 | 0.0398 | 0.0503 | -121.33 | | 106.40 | 148.47 | 98.72 | 0.0109 | | 0.0547 | 0.0933 | 0.1987 |
|  | 3 | 0.0025 | 0.0306 | 0.0042 | 0.0135 | -0.0080 | | 0.0410 | 0.0088 | 0.0262 | 0.0373 | | 0.0351 | 0.0246 | 0.0178 | -130.14 | | 107.57 | 62.39 | 78.48 | 0.0463 | | 0.1216 | 0.0324 | 0.0550 |
|  | 13 | -0.0057 | 0.0229 | 0.0116 | 0.0168 | 0.0032 | | 0.0310 | -0.0142 | 0.0510 | 0.0300 | | 0.0243 | 0.0411 | 0.0378 | -131.47 | | 121.42 | 141.72 | 100.32 | 0.0228 | | 0.0594 | 0.1066 | 0.1573 |
|  | 14 | -0.0013 | 0.0194 | 0.0158 | 0.0164 | 0.0003 | | 0.0261 | -0.0169 | 0.0442 | 0.0256 | | 0.0193 | 0.0342 | 0.0392 | -2.76 | | 122.56 | 165.52 | 77.95 | 0.0077 | | 0.0569 | 0.0954 | 0.1565 |
|  | 15 | -0.0010 | 0.0244 | 0.0090 | 0.0169 | -0.0028 | | 0.0273 | -0.0184 | 0.0391 | 0.0295 | | 0.0209 | 0.0331 | 0.0330 | -140.97 | | 90.71 | 164.79 | 89.06 | 0.0234 | | 0.0673 | 0.0827 | 0.1386 |
|  | Mean | 0.0001 | 0.0241 | 0.0091 | 0.0208 | -0.0041 | | 0.0362 | -0.0102 | 0.0459 | 0.0321 | | 0.0296 | 0.0372 | 0.0370 | -124.04 | | 104.84 | 141.94 | 91.52 | 0.0316 | | 0.0952 | 0.0863 | 0.1405 |
| Pre-CIT2 | 1 | 0.0073 | 0.0291 | 0.0058 | 0.0317 | -0.0216 | | 0.0611 | -0.0123 | 0.0527 | 0.0433 | | 0.0562 | 0.0472 | 0.0398 | 200.22 | | 87.22 | 143.70 | 110.11 | 0.0752 | | 0.2274 | 0.1091 | 0.1488 |
|  | 2 | -0.004 | 0.017 | 0.012 | 0.0206 | -0.0002 | | 0.0291 | -0.0133 | 0.0609 | 0.0266 | | 0.0203 | 0.0384 | 0.0537 | 201.13 | | 107.13 | 147.58 | 78.18 | 0.0079 | | 0.0545 | 0.0963 | 0.2110 |
|  | 3 | 0.0007 | 0.03 | 0.0054 | 0.0125 | -0.0108 | | 0.0405 | 0.0072 | 0.0266 | 0.0369 | | 0.0351 | 0.0250 | 0.0167 | 104.74 | | 137.60 | 94.22 | 89.11 | 0.0433 | | 0.1255 | 0.0399 | 0.0516 |
|  | 13 | -0.0082 | 0.0219 | 0.0122 | 0.0188 | 0.0026 | | 0.0319 | -0.0135 | 0.0502 | 0.0299 | | 0.2532 | 0.0427 | 0.0356 | 138.51 | | 140.17 | 131.47 | 92.39 | 0.0073 | | 0.0539 | 0.1054 | 0.1517 |
|  | 14 | -0.0039 | 0.0184 | 0.0163 | 0.0179 | -0.0002 | | 0.0263 | -0.0173 | 0.0478 | 0.0254 | | 0.0192 | 0.0374 | 0.0414 | 108.55 | | 137.59 | 160.31 | 76.67 | 0.0055 | | 0.0586 | 0.1018 | 0.1680 |
|  | 15 | -0.0024 | 0.0244 | 0.0089 | 0.0184 | -0.0043 | | 0.0291 | -0.0199 | 0.041 | 0.0305 | | 0.0221 | 0.0354 | 0.0342 | 195.45 | | 103.41 | 173.55 | 90.58 | 0.0215 | | 0.0743 | 0.0996 | 0.1421 |
|  | Mean | -0.0018 | 0.0235 | 0.0101 | 0.0200 | -0.0058 | | 0.0363 | -0.0115 | 0.0465 | 0.0321 | | 0.0677 | 0.0377 | 0.0369 | 158.89 | | 118.98 | 142.43 | 89.46 | 0.0268 | | 0.0990 | 0.0920 | 0.1455 |
| Pre-Post | 1 | 0.001 | 0.0521 | -0.0266 | 0.0624 | -0.0342 | | 0.0575 | 0.013 | 0.097 | 0.0630 | | 0.0556 | 0.0883 | 0.0765 | 205.39 | | 89.00 | -58.01 | 101.45 | 0.0519 | | 0.2304 | 0.0781 | 0.1712 |
|  | 2 | -0.0161 | 0.0446 | -0.007 | 0.0355 | -0.0198 | | 0.0806 | -0.0055 | 0.0328 | 0.0632 | | 0.0706 | 0.0411 | 0.0246 | 208.93 | | 111.19 | -63.31 | 105.02 | -0.0140 | | 0.1942 | 0.0159 | 0.0953 |
|  | 3 | -0.0157 | 0.0477 | -0.0222 | 0.0348 | -0.0065 | | 0.0681 | 0.0314 | 0.0409 | 0.0725 | | 0.0413 | 0.0547 | 0.0354 | 181.29 | | 10.79 | 137.86 | 63.72 | 0.0550 | | 0.1994 | 0.0081 | 0.0539 |
|  | 13 | -0.0259 | 0.0509 | 0.007 | 0.0461 | 0.0025 | | 0.038 | -0.0455 | 0.0819 | 0.0542 | | 0.0408 | 0.0687 | 0.0776 | 46.23 | | 140.97 | -133.89 | 76.71 | -0.0089 | | 0.0833 | 0.1081 | 0.3274 |
|  | 14 | -0.0118 | 0.0454 | 0.0058 | 0.0304 | -0.0228 | | 0.0395 | -0.0315 | 0.035 | 0.0506 | | 0.0404 | 0.0452 | 0.0324 | 39.78 | | 132.15 | 218.12 | 218.12 | 0.0240 | | 0.1376 | 0.0721 | 0.1584 |
|  | 15 | -0.0213 | 0.041 | -0.0025 | 0.0388 | -0.0129 | | 0.0464 | -0.0287 | 0.0419 | 0.0564 | | 0.0340 | 0.0492 | 0.0394 | 213.09 | | 108.12 | -121.01 | 71.21 | -0.0354 | | 0.1038 | 0.0689 | 0.1746 |
|  | Mean | -0.0150 | 0.0470 | -0.0076 | 0.0413 | -0.0156 | | 0.0550 | -0.0111 | 0.0549 | 0.0600 | | 0.0471 | 0.0579 | 0.0477 | 183.16 | | 104.16 | -118.29 | 93.45 | 0.0121 | | 0.1581 | 0.0585 | 0.1635 |

This analysis was done with healthy controls (N = 22) and patients with ABI (N = 15). COE: changes in cerebral oxygen exchange, CBV: changes in total blood volume in the brain tissue, L: the length of the vector, k: the degree of the vector, PRI: phase-associated response intensity.

**Table 5. Demographic Characteristics of Patients with ABI (n = 15)**

| ID | Age | Gender | Lesion Area | K-MMSE |
| --- | --- | --- | --- | --- |
|  | | | Cortical lesions excluding prefrontal cortex |  |
| 1 | 62 | M | Right middle cerebral artery infarction | 26 |
| 2 | 31 | F | Left middle cerebral artery infarction | 24 |
| 3 | 64 | M | Left middle cerebral artery infarction | 28 |
| 4 | 39 | M | Left middle cerebral artery infarction | 24 |
| 5 | 45 | M | Right middle cerebral artery infarction | 30 |
|  | | | Intracranial lesions |  |
| 6 | 69 | M | Subdural hemorrhage, subarachnoid hemorrhage | 29 |
| 7 | 57 | M | Subarachnoid hemorrhage | 30 |
| 8 | 62 | M | Subdural hemorrhage | 19 |
| 9 | 52 | M | Epidural hemorrhage | 18 |
| 10 | 56 | M | Subdural hemorrhage | 30 |
| 11 | 54 | M | Subarachnoid hemorrhage | 23 |
|  | | | Subcortical lesions |  |
| 12 | 45 | M | Intraventricular hemorrhage | 29 |
| 13 | 57 | M | Intraventricular hemorrhage | 20 |
| 14 | 56 | F | Left basal ganglia intracerebral hemorrhage | 24 |
| 15 | 37 | M | Hypoxic brain injury | 29 |

K-MMSE: Korean version of the Mini-Mental State Examination

**Table 6.** Phase classification of initial dip phases haemodynamic changes^82-83, 124, 126-127^

|  | Phase | Relationships among indices | Brain activity |
| --- | --- | --- | --- |
| 1 | Hyperoxia/Hyperemia | 0 < ΔHHb < ΔHbO_2_, ΔCOE < 0 < ΔCBV | Increased brain activity in relation to oxygen exchange |
| 2 | Hypoxia/Hyperemia | 0 < ΔHbO_2_ < ΔHHb, 0 < ΔCOE < ΔCBV |  |
| 3 | Hypoxia/Hyperemia | ΔHbO_2_ < 0 < ΔHHb, 0 < ΔCBV < ΔCOE |  |
| 4 | Hypoxia/Ischemia | ΔHbO_2_ *<* 0 < ΔHHb, ΔCBV < 0 < ΔCOE |  |
| 5 | Hypoxia/Ischemia | ΔHbO_2_ < ΔHHb *<* 0, ΔCBV < 0 < ΔCOE |  |
| -1 | Hyperoxia/Hyperemia | ΔHHb < 0 *<* ΔHbO_2_, ΔCOE < 0 < ΔCBV | No increases in brain activity in relation to oxygen exchange |
| -2 | Hyperoxia/Ischemia | ΔHHb < 0 *<* ΔHbO_2_, ΔCOE < ΔCBV < 0 |  |
| -3 | Hyperoxia/Ischemia | ΔHHb *<* ΔHbO_2_ < 0, ΔCBV< ΔCOE < 0 |  |
